# Comparison of Direct and Indirect Laryngoscopes in Vomitus and Hematemesis Settings: A Randomized Simulation Trial

**DOI:** 10.1155/2015/806243

**Published:** 2015-11-05

**Authors:** Ryosuke Mihara, Nobuyasu Komasawa, Sayuri Matsunami, Toshiaki Minami

**Affiliations:** Department of Anesthesiology, Osaka Medical College, 2-7 Daigaku-machi, Takatsuki, Osaka, Japan

## Abstract

*Background.* Videolaryngoscopes may not be useful in the presence of hematemesis or vomitus. We compared the utility of the Macintosh laryngoscope (McL), which is a direct laryngoscope, with that of the Pentax-AWS Airwayscope (AWS) and McGRATH MAC (McGRATH), which are videolaryngoscopes, in simulated hematemesis and vomitus settings.* Methods.* Seventeen anesthesiologists with more than 1 year of experience performed tracheal intubation on an adult manikin using McL, AWS, and McGRATH under normal, hematemesis, and vomitus simulations.* Results.* In the normal setting, the intubation success rate was 100% for all three laryngoscopes. In the hematemesis settings, the intubation success rate differed significantly among the three laryngoscopes (*P* = 0.021). In the vomitus settings, all participants succeeded in tracheal intubation with McL or McGRATH, while five failed in the AWS trial with significant difference (*P* = 0.003). The intubation time did not significantly differ in normal settings, while it was significantly longer in the AWS trial compared to McL or McGRATH trial in the hematemesis or vomitus settings (*P* < 0.001, compared to McL or McGRATH in both settings).* Conclusion.* The performance of McGRATH and McL can be superior to that of AWS for tracheal intubation in vomitus and hematemesis settings in adults.

## 1. Background

The European Resuscitation Council (ERC) cardiopulmonary resuscitation (CPR) guidelines emphasize the importance of rapid and definite tracheal intubation [1]. The guidelines also suggest that skilled rescuers should be able to secure the airway without interrupting chest compressions to visualize the vocal cords and allow the passage of the tracheal tube [2].

The Pentax Airwayscope (AWS; Hoya, Tokyo, Japan) is a videolaryngoscope reported to provide an indirect view of the airway [3]. Studies indicate that AWS is useful not only for difficult airway management but also for emergent tracheal intubation during resuscitation by simulation analysis [4, 5]. The McGRATH (McGRATH; Aircraft Medical Ltd., Edinburgh, UK) is a device that has been developed with a high-resolution video camera, providing direct and indirect views of the glottis, and is reportedly useful for intubating several difficult airways [6]. While AWS and McGRATH are both considered convenient tools for difficult or emergent airway management, their indirect monitors may not be useful in the presence of hematemesis or vomitus in the pharynx [7]. In such patients, direct laryngoscopes such as the Macintosh laryngoscope (McL) may be superior to videolaryngoscopes for definite tracheal intubation.

The utility of direct (McL) and indirect laryngoscopes (AWS and McGRATH) for tracheal intubation has not yet been validated; therefore, we decided to compare the utility of McL with that of AWS and McGRATH in hematemesis and vomitus settings. Because direct clinical evaluation is unethical, we hypothesized that AWS and McGRATH would improve intubation in simulated hematemesis and vomitus settings and compared them with McL in terms of the ease of tracheal intubation by nonanesthesiologists using an adult manikin with hematemesis and vomitus simulations.

## 2. Methods

From November to December 2014, 17 doctors with more than a year of experience in anesthesiology or critical care medicine were recruited from medical personnel taking an airway management or sedation training course at the Osaka Medical College. Written informed consent was obtained before the study. This study was approved by the Osaka Medical College Research Ethics Committee (Approval number 1321).

The Airway Trainer (Laerdal, Stavanger, Norway), designed to accurately represent an adult male, was used for the study simulations and intubations. Participants used a tracheal tube (Portex, St. Paul, MN, USA) with an internal diameter of 7.5 mm.

**Figure 1:**
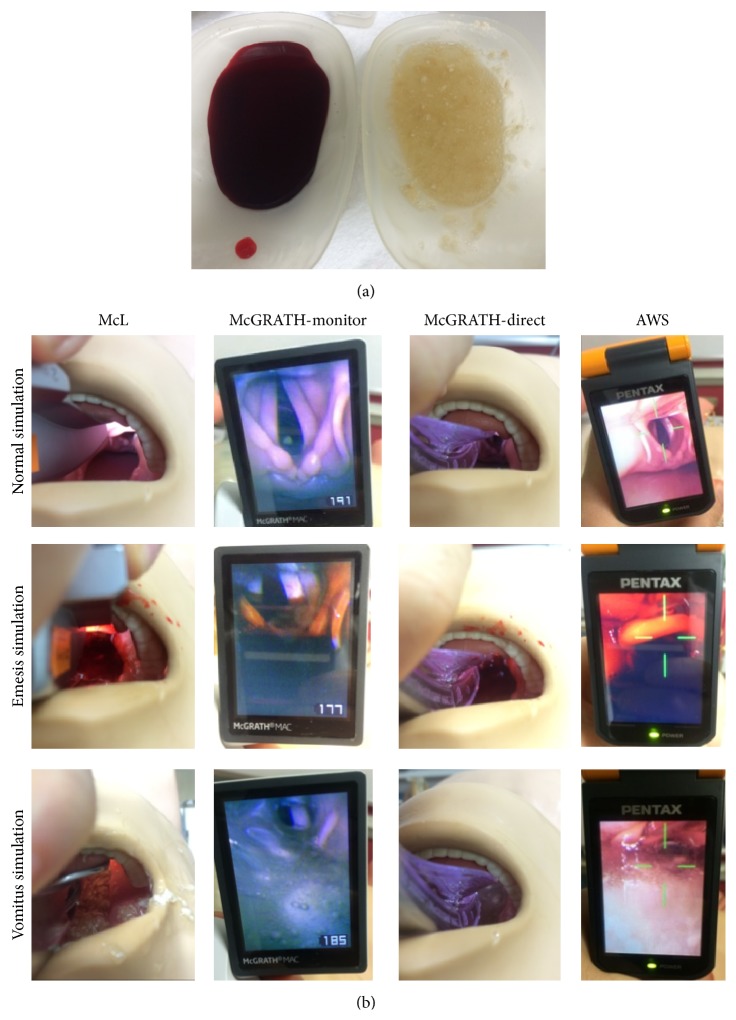
Simulated hematemesis and vomitus settings. (a) Simulated hematemesis and vomitus. (b) A representative laryngoscopic view with three laryngoscopes in each simulation condition (normal, hematemesis, and vomitus).

Simulated stomach contents (vomitus; simulated stomach contents, Laerdal, Norway) or simulated blood (hematemesis; simulated blood, Kyoto-Kagaku, Japan) were added to the pharynx of the manikin. The contents were prepared by dissolving 10 g of powder in 200 mL of water according to the manufacturer's instructions and were poured into the pharynx to the level of covering the epiglottis to simulate vomitus or hematemesis. The lower esophagus was clamped with forceps to keep these liquids in the pharynx. We also clamped both bronchi instead of the trachea because clamping the trachea impedes smooth tracheal intubation. The different views (normal, hematemesis, and vomitus setting) of the three devices are shown in Figure 1.

The manikin was placed on a hard, flat table for “on the resuscitation bed” simulation. Each participant was instructed to insert the tracheal tube with the three laryngoscopes (McL, AWS, and McGRATH), attach a bag valve mask, and attempt to ventilate the lungs of the manikin. In McL and McGRATH trials, participants used size 4 blade. In AWS trial, standard Introck (ITL-SL, HOYA, Japan) was used. Participants were given 10 min to practice intubation, with the instructor available for advice. The appropriate equipment for each trial was placed in a box next to the manikin's head. Intubation started when the participant picked up McL, AWS, or McGRATH and ended at the point of manual ventilation after tube insertion. The number of intubation sessions was recorded for both tracheal and esophageal intubations. At the end of the study, participants rated the difficulty of using each device using a visual analog scale (VAS) from 0 mm (extremely easy) to 100 mm (extremely difficult).

Statistical analysis was performed utilizing JMP 11 (SAS Institute Inc., Cary, NC, USA). Results obtained from each trial were compared using one-way repeated measures analysis of variance for intubation time and VAS and chi-square test for the success rate. Data are presented as means ± standard deviations (SDs). A *P* value of < 0.05 was considered statistically significant.

The study was designed as a randomized crossover trial to minimize the order effect. In each McL, AWS, and McGRATH trial, participants performed tracheal intubation in all three simulations (normal, hematemesis, and vomitus). The order of intervention was randomized for each participant using the random number table, resulting in a total of nine interventions per participant.

The sample size was calculated on the basis of our preliminary study on the time required for intubation with McL and McGRATH in the vomitus setting in eight participants. The mean (SD) time was 11.1 ± 3.3 s for McL and 5.9 ± 3.6 s for McGRATH. Using an *α* error of 0.05 and a *β* error of 0.2, we estimated that 15 participants would be adequate for each group. Therefore, we planned to recruit 17 participants for each group to adjust for missing data.

## 3. Results

The mean clinical experience of the 17 participants (11 male, 6 female) was 6.4 ± 3.6 years. The number of times the participants had worked before participating in the trial with McL, AWS, and McGRATH was 911.8 ± 501.1, 128.8 ± 94.2, and 68.2 ± 66.0, respectively.

### 3.1. Endotracheal Intubation Success with McL, AWS, and McGRATH

The number of successful tracheal intubations for each device is displayed in Table 1. In McL or McGRATH trial, the intubation success rate did not significantly differ among the three settings (*P* = 0.125 for McL trial, *P* = 0.361 for McGRATH trial). Contrastingly, in the AWS trial, the intubation success rate differed significantly among the simulated situations (*P* < 0.001).

In the normal setting, the intubation success rate was 100% for all three laryngoscopes. In the hematemesis settings, the intubation success rate differed significantly among the three laryngoscopes (*P* = 0.021). In the vomitus settings, all participants succeeded in tracheal intubation with McL or McGRATH, while five failed in the AWS trial with significant difference (*P* = 0.003).

### 3.2. Intubation Time with McL, AWS, and McGRATH

**Figure 2:**
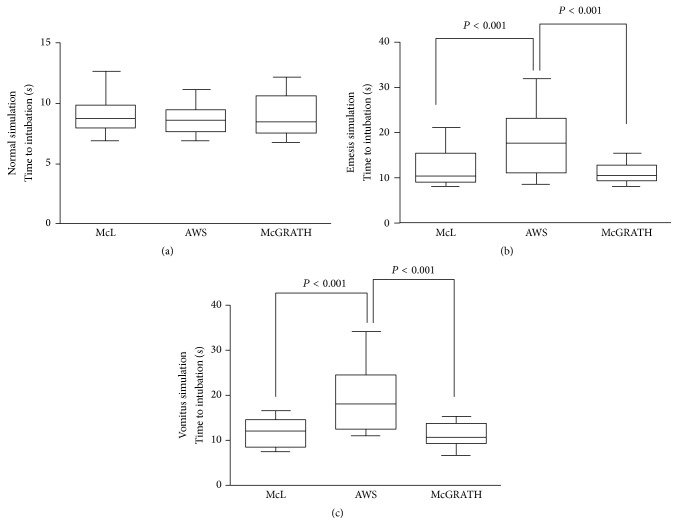
Box-and-whisker plot (median, IQR, and range) of time required for simulated tracheal intubation in hematemesis and vomitus settings using each laryngoscope. Results are expressed as means ± SDs and were analyzed using one-way analysis of variance. (a) Normal setting, (b) hematemesis, and (c) vomitus setting. AWS, Pentax-AWS Airwayscope; McGRATH, McGRATH MAC; McL, Macintosh laryngoscope.

The intubation time in each setting is shown in Figure 2. In the normal setting, the intubation time did not differ significantly among the three laryngoscopes, while in the hematemesis and vomitus settings the intubation time was significantly longer with AWS than with McL and McGRATH (*P* < 0.001, compared to McL or McGRATH in both settings). There was no significant difference in the intubation time between McL and McGRATH in both the hematemesis and the vomitus settings.

### 3.3. VAS Scores for Difficulty of Tracheal Intubation with McL, AWS, and McGRATH

**Figure 3:**
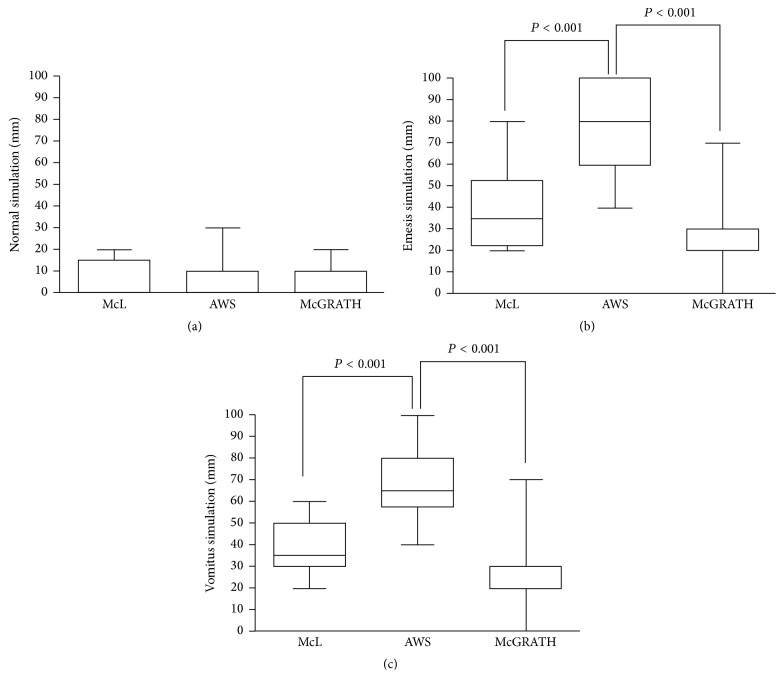
Box-and-whisker plot (median, IQR, and range) of visual analog scale scores for difficulty of simulated tracheal intubation in hematemesis and vomitus settings using each laryngoscope. (a) Normal setting, (b) hematemesis setting, and (c) vomitus setting. Results are expressed as means ± SDs and were analyzed using one-way analysis of variance. AWS, Pentax-AWS Airwayscope; McGRATH, McGRATH MAC; McL, Macintosh laryngoscope.

As shown in Figure 3, the subjective difficulty of tracheal intubation did not differ in the normal setting, while it was significantly higher with AWS than with McL and McGRATH in the hematemesis and vomitus settings (*P* < 0.001, compared to McL or McGRATH in both settings). The VAS score was not significantly different between McL and McGRATH in both the hematemesis and vomitus settings.

## 4. Discussion

Airway management is considered an essential element, particularly for in-hospital CPR. While conventional direct-view laryngoscopes such as McL are the most widely used for tracheal intubation, it is difficult to master the skills required for use, and the incidence of inaccurate intubation can be unacceptably high for occasional operators [8, 9]. There are several reasons for the difficulty in airway management during resuscitation, such as chest compression, position of the rescuer or victim, and restriction of the airway management device [10]. A major problem encountered during airway management is vomitus or blood in the pharynx and neck fixation with the cervical collar. A nonnegligible number of patients exhibit vomiting or hematemesis during sudden cardiac arrest, leading to difficulty in tracheal intubation during resuscitation [11, 12]. In such situations, Easy Tube or combitube may be safe against aspiration and can therefore be used in vomiting or bleeding patients unless the glottis cannot be visualized. However, these devices usually do not complete protection of the trachea, and definite tracheal intubation is preferable in some circumstances [2].

Pentax-AWS Airwayscope is a videolaryngoscope for tracheal intubation designed to provide a clear view of the glottis and its surrounding structures. It improves the laryngeal view, and its tube guide facilitates rapid and reliable tracheal intubation under vision, even in difficult situations such as cervical neck immobility or morbid obesity [3]. Increasing evidence indicates that AWS is suitable for tracheal intubation during various difficult airway management and simulated emergency situations [13]. However, one clinical study showed that AWS did not show superiority to McL in prehospital settings, as opposed to simulated in-hospital situations [14]. We speculate that vomitus or hematemesis may have contributed to the lower success rate of AWS in the prehospital situations.

McGRATH is a portable videolaryngoscope that provides excellent laryngoscopic views in patients with normal airways and patients in whom direct laryngoscopy is difficult or fails [15]. While AWS provides only an indirect view of the glottis, McGRATH provides both direct and indirect views. There are several reports on the utility of these devices for airway management during resuscitation.

The present study found that the intubation time with AWS was significantly longer in the vomitus and hematemesis settings than in the normal setting, accompanied by a significant intubation success rate difference. In contrast, the intubation time did not significantly increase with McL and McGRATH. Only one or two participants failed in the hematemesis setting and all anesthesiologists were successful in intubating in the vomitus settings with McL or McGRATH. One probable reason for the difficulties experienced with AWS is that the video monitor is severely disturbed by vomitus and hematemesis. In contrast, as McGRATH can provide not only an indirect video monitor view but also a direct laryngeal view, participants could perform definite tracheal intubation, even in vomitus and hematemesis settings. Furthermore, because McGRATH provides a better laryngeal view compared with the conventional McL [15], it may be useful for emergent tracheal intubation in vomitus and hematemesis settings.

This study has several limitations. First, intubation was performed on a manikin, which leads to shorter airway intervention times than those required for actual patients [16, 17]. Second, the use of these three devices may be less than ideal in patients with difficult airways, such as those with a severely restricted mouth opening or a small jaw. Third, the simulations do not account for other factors related to resuscitation, such as chest compression and cervical stabilization [18]. Finally, the homogeneity of hematemesis and vomitus cannot completely simulate clinical situations.

For future directions, there is a controversy whether securing the airway using tracheal intubation is the best way of airway management during out-of-hospital cardiac arrest [7, 19]. Thus, in the next study, it may be interesting to evaluate the efficacy of supraglottic devices such as laryngeal mask, laryngeal tube, or combitube for airway management in the hematemesis or vomitus settings.

## 5. Conclusion

Within the limitations of our study, we conclude McGRATH and McL can show superior performance compared with AWS for tracheal intubation in adults with vomitus or blood in the pharynx. Future studies examining accumulated evidence of clinical experiences and randomized trials of McL, AWS, and McGRATH in actual patients with hematemesis or vomitus are required to clarify the findings of this simulation study.

## Figures and Tables

**Table 1:** Tracheal intubation success rates for McL, AWS, and McGRATH in normal, hematemesis, and vomitus settings.

	Normal simulation (successful/total)	Hematemesis simulation (successful/total)	Vomitus simulation (successful/total)	*P* value
McL	17/17	15/17	17/17	0.125
AWS	17/17	10/17	12/17	<0.001
McGRATH	17/17	16/17	17/17	0.361
*P* value	0.978	0.021	0.003	

AWS, Pentax-AWS Airwayscope; McGRATH, McGRATH MAC; McL, Macintosh laryngoscope.

Numerator: number of participants who were successfully intubated.

Denominator: number of participants for whom tracheal intubation was attempted.

Differences were analyzed using chi-square test.

## Abbreviations

CPR: Cardiopulmonary resuscitation
ERC: European Resuscitation Council
AWS: Pentax-AWS Airwayscope
McGRATH: McGRATH MAC
McL: Macintosh laryngoscope.
